# p27 Is a Critical Prognostic Biomarker in Non-Alcoholic Steatohepatitis-Related Hepatocellular Carcinoma

**DOI:** 10.3390/ijms141223499

**Published:** 2013-11-29

**Authors:** Yasunobu Matsuda, Toshifumi Wakai, Yuki Hirose, Mami Osawa, Shun Fujimaki, Masayuki Kubota

**Affiliations:** 1 Department of Medical Technology, Niigata University Graduate School of Health Sciences, 2-746 Asahimachi-dori, Chuo-Ku, Niigata 951-8518, Japan; E-Mail: b10m206b@alumni.niigata-u.ac.jp; 2 Division of Digestive and General Surgery, Niigata University Graduate School of Medical and Dental Sciences, 1-757 Asahimachi-dori, Chuo-Ku, Niigata 951-8510, Japan; E-Mails: wakait@med.niigata-u.ac.jp (T.W.); y-hirose@med.niigata-u.ac.jp (Y.H.); 3 Department of Pediatric Surgery, Niigata University Graduate School of Medical and Dental Sciences, 1-757 Asahimachi-dori, Chuo-Ku, Niigata 951-8510, Japan; E-Mails: mamix.3211@gmail.com (M.O.); mkubota@med.niigata-u.ac.jp (M.K.)

**Keywords:** p27, nonalcoholic steatohepatitis, hepatocellular carcinoma, prognosis

## Abstract

Non-alcoholic steatohepatitis (NASH) is a recently identified chronic liver disease, which progresses to liver cirrhosis and hepatocellular carcinoma (HCC). As the number of patients studied to date has been limited, clinically useful prognostic biomarkers of NASH-related HCC have not been available. In this study, we investigated the status of a cell-cycle regulator, p27, in NASH-related HCC. p27 has been regarded as a prognostic factor in various types of cancer patients. A total of 22 cases with NASH-related HCC were analyzed for p27 protein expression, and phosphorylation at threonine 157 (T157) and serine 10 (S10) by immunohistochemical analysis. The correlation of p27 with tumor characteristics, disease-free survival (DFS), and overall survival was analyzed. p27 expression was decreased in 13 HCCs (59%), and was significantly correlated with enlarged tumor size (*p =* 0.01) and increased cell proliferation (*p <* 0.01). Phospho-p27 at T157 and S10 was detected in four (18%) and seven (32%) cases, respectively, and patients positive for phospho-p27 (S10) showed reduced DFS (hazard ratio 7.623, *p =* 0.016) by univariate analysis. Further studies with more patients are required to verify the usefulness of p27 as a biomarker for predicting tumor recurrence in NASH patients.

## 1. Introduction

Hepatocellular carcinoma (HCC) is one of the most common malignant cancers in the world, the incidence of which is rising in many places around the world, including Africa, China, and South East Asia [1–3]. Surgical resection of the tumors provides adequate survival for patients with HCC, but their outcome remains unsatisfactory owing to early metastasis and recurrence after treatment [4–7]. HCC is etiologically unique in that it arises from various types of chronic hepatic diseases such as infection with hepatitis B or C virus (HBV, HCV), and alcohol abuse. Recently, nonalcoholic steatohepatitis (NASH), an advanced type of the obese-related nonalcoholic fatty liver disease (NAFLD), has been recognized as another causative agent of HCC. NASH is a newly identified, non-viral chronic liver disease, which can progress to liver cirrhosis, accompanied by liver failure and HCC, and the prevalence of NASH-related HCC has been increasing in developing countries [1,3,5]. Unfortunately, the precise reason for cancer development in NASH patients has remained enigmatic and clinically useful biomarkers, predicting the risk and the prognosis of NASH-related HCC, have not been discovered to date.

The mechanism of cancer progression has been generally considered to be a serial process of cellular events involving enhanced cell proliferation, loss of cell-cell contact, cell migration, degradation of extracellular matrix, and enhanced cell proliferation at the seeded sites [8]. Many studies have suggested that deregulated cell-cycle regulators are deeply involved in this process, and examining the status of cell-cycle regulators might be valuable for predicting the prognosis of cancer patients. Among several types of cell-cycle regulators, the G1/S cell-cycle inhibitor p27 has been proposed as a critical determinant of tumor aggressiveness in hepatitis virus-related HCC [9,10]. Clinicopathological studies have reported that loss of p27 is frequently observed in cancer patients with poor prognosis, suggesting that p27 might be an adverse prognostic indicator in hepatitis virus-related HCCs [9–14]. p27 is a member of the KIP family of kinase inhibitors that regulate the kinase activities of the cyclin-dependent kinase 2 (CDK2)/cyclin A and CDK2/cyclin E complexes. p27 knock-out mice show oncogenic cellular proliferation and cell enlargement due to the hyper-activation of CDK2 kinase in multiple organs [15,16], indicating that loss of p27 strongly drives tumorigenesis. It should be noted, however, the function of p27 is not only defined by the level of protein expression but also by protein phosphorylation [17,18]. Some sets of aggressive cancer cells, for example, contain p27 that has been inactivated by Akt-mediated phosphorylation at threonine 157 (T157) and is anomalously overexpressed in the cytoplasm [19–21]. We and other investigators have reported that cytoplasmic expression of p27 in HBV- and HCV-related HCCs, possibly due to the phosphorylation at Thr157, positively correlated with the degree of cellular proliferation and tumor invasion [22–24]. More recently, it was shown that p27 is phosphorylated at S10 by kinase-interacting stathmin (KIS) [25] and co-localizes with actin to induce enhanced cell migration [26,27]. This is independent from its function in inhibiting cell-cycle progression, and these results suggest that evaluating both the levels of protein expression and phosphorylation might help to improve the clinical usefulness of p27. In this study, we thoroughly investigated the status of p27, including the level of protein expression and the phosphorylation levels at T157 and S10, in NASH-related HCCs by immunohistochemical analysis. To investigate the possible contribution of p27 to tumor behavior, the clinicopathological features of patients with NASH-related HCC were compared with their prognosis according to the status of p27.

## 2. Results and Discussion

### 2.1. Results

#### 2.1.1. Patients Characteristics

Twenty-two patients with NASH-related HCC (12 men and 10 women, mean age 71 ± 7 years, range: 55–83 years), who had undergone curative surgery between 1998 and 2011 at the Niigata Medical University Hospital in Japan, were retrospectively enrolled for the study. All the cases were serologically negative for both the HBV surface antigen and the HCV antibody. Follow-up data of the postoperative outcomes were retrospectively obtained from all the patients and the follow-up period was in the range of 1.5–154.0 months (mean, 36.5 months). Profiles of the tumor characteristics obtained from the cases are summarized in Table 1.

#### 2.1.2. Expression Profile of p27 and Phospho-p27 in NASH-Related HCC

**Figure 1.**
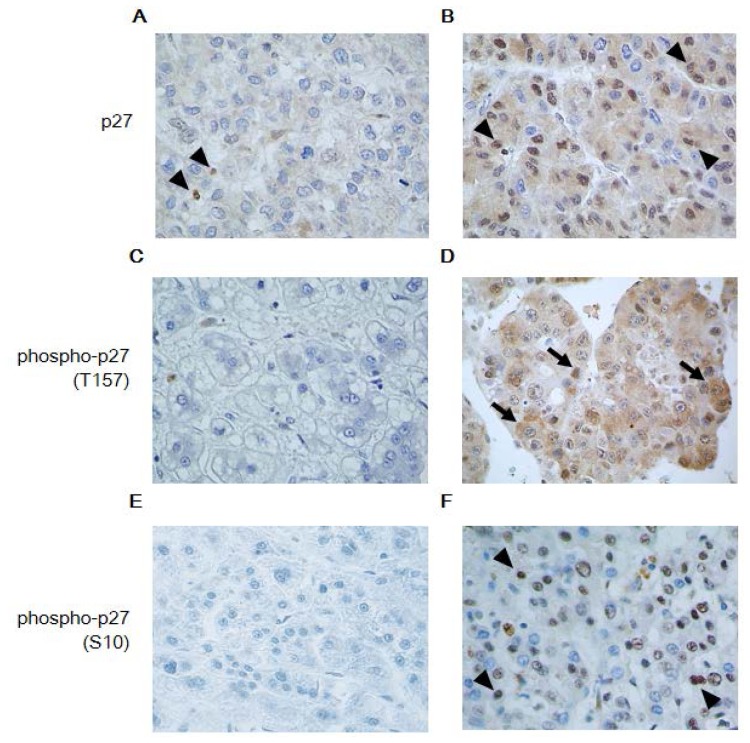
Representative immunohistochemical staining of p27, phospho-27 (T157), and phospho-p27 (S10) in NASH-related HCC tissues. Negative and positive immunostaining for p27 (**A** and **B**), phospho-p27 (T157) (**C** and **D**), and phospho-p27 (S10) (**E** and **F**) in NASH-related HCC tissue samples. Positive immunostaining for p27 and phospho-p27 (S10) were localized in the nuclei (arrowhead), while phospho-p27 (T157) was expressed in either nuclei or cytoplasm (arrow) (original magnification: ×100).

In normal liver tissue samples, p27 was widely expressed in the nuclei of hepatocytes as previously reported [14,28], while phospho-p27 (T157 and S10) was not detected by immunohistochemical analysis (not shown). In cases with NASH-related HCC, labeling indices (L.I.) of p27 was less than <50% in 13 of 22 (59%) cases (low-p27 expressers; Figure 1A), while nine (41%) cases expressed above 50% of the p27 L.I. in the tumors (high-p27 expressers; Figure 1B). Cytoplasmic and nuclear expression of phospho-p27 (T157) was observed in 4 of 22 HCCs (18%) (Figure 1C,D). Phospho-p27 (S10) was detected in the nuclei of seven HCCs (32%), with intense nuclear immunoreactivity (Figure 1E,F). All the cases with positive-phospho-p27 (T157) were included within a group of high-p27 expressers (Spearman rank correlation test: *r =* 0.652, *p =* 0.002; Table 2), while the level of p27 expression was not statistically correlated with phospho-p27 (S10) in NASH-related HCC (*r =* 0.193, *p =* 0.378; Table 2).

**Figure 2.**
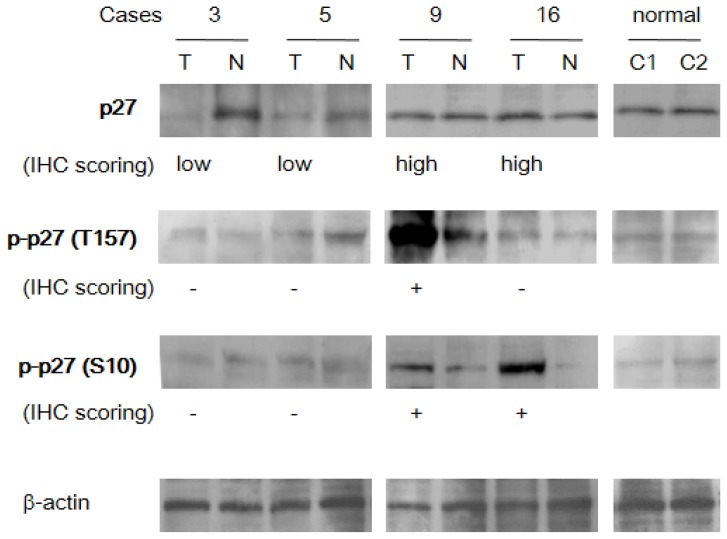
Analysis of p27 expression in HCC in representative tissue samples by western blotting. Numbers represent tissue samples obtained from NASH patients with HCC. The results of immunohistochemical staining (IHC scoring) in each sample are shown at the bottom of each lane. T, tumor tissues; N, adjacent non-tumorous tissue for each HCC sample; normal, normal liver (C1 and C2; normal cases 1 and 2).

The results of Western blotting of tissue samples correlated well with the results of immunohistochemical analysis. p27 was strongly expressed in normal liver tissue samples, while phosphorylated p27 was faint at T157 and S10. In HCC samples, a protein band corresponding to p27 was faint in two out of two cases identified with low p27 expression by immunohistochemical analysis (Figure 2; cases 3 and 5). In contrast, protein band intensities revealed a 0.8 to 1.2-fold increase over adjacent non-HCC liver tissues in two out of two HCC samples, in which high p27 expression was detected by immunohistochemistry (case 9 and 16). P-p27 (T157) and p-p27 (S10) were strongly detectable by Western blotting in case 9 and, both, 9 and 16, respectively, in which phosphorylated p27 were detected by immunohistochemical analysis.

#### 2.1.3. Association between p27 and Cell Proliferation in NASH-Related HCC

**Figure 3.**
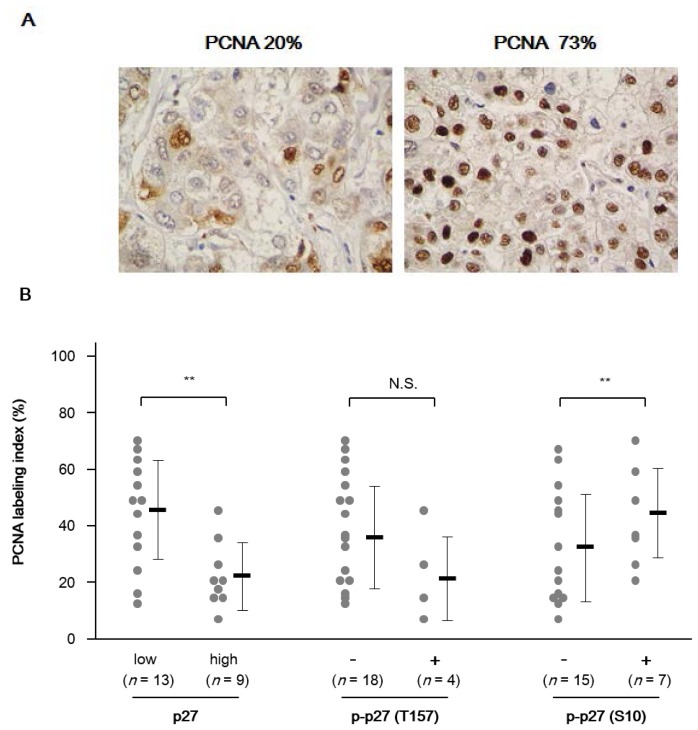
Correlation between the PCNA labeling index and p27 status in NASH-related HCC. (**A**) Representative images of immunostaining for PCNA in HCC (original magnification: ×100); and (**B**) Dot plots of the labeling index of PCNA in the subgroups according to different expression of p27 and phospho-p27. The horizontal bar depicts the mean value and the vertical bars indicate the standard deviation. *p*-values were obtained by the Mann-Whitney test. N.S., not significant; **p < 0.01.

The results of immunostaining for proliferation cell nuclear antigen (PCNA) showed that L.I. of PCNA was in the range of 8%–73% in NASH-related HCCs, indicating that the cell proliferation behavior of the tumors varied among the cases (Figure 3A). Mean levels of L.I. of PCNA were 44.5% ± 18.3% and 22.4% ± 12.0% in low- and high-p27 expressers (Mann-Whitney test, *p =* 0.009), respectively, indicating that the level of p27 expression was inversely correlated with the cell proliferation in the tumors (Figure 3B). When the subgroups were compared according to the level of phospho-p27 (T157), mean L.I. of PCNA was 22.5% ± 16.3% and 38.4% ± 19.0% (*p =* 0.136) in phospho-p27 (T157)-negative and -positive groups, respectively, indicating that p27 phosphorylation at this site does not correlate with the cell proliferation. In contrast, mean levels of PCNA L.I. in phospho-p27 (S10)-negative and -positive groups were 32.2% ± 19.5% and 42.6% ± 18.1% (*p* < 0.001) (Figure 3B), respectively, indicating that the phosphorylation of p27 at S10 is significantly correlated with cell proliferation in NASH-related HCC.

#### 2.1.4. Correlation of p27, Phospho-p27 (T157), and Phospho-p27 (S10) Expression with Clinicopathological Profiles in HCC

The frequencies of p27 expression, phospho-p27 (T157), and phospho-p27 (S10) in relation to different clinicopathological parameters are shown in Table 3. A decrease in p27 expression was significantly associated with tumor size (*p =* 0.010), and although not statistically different, cases of low-p27 expressers showed an increased rate of vascular invasion (*p =* 0.316), progressive histological grade (*p =* 0.155), and tumor stage advancement (*p =* 0.135). The presence of phospho-p27 (T157) was significantly correlated with advanced histological grade (*p =* 0.045). The level of phospho-p27 (S10) was not significantly correlated with any of the clinicopathological features of HCC.

#### 2.1.5. Prognostic Value of the Combination of p27 and Phospho-p27 (S10) in HCC

**Figure 4.**
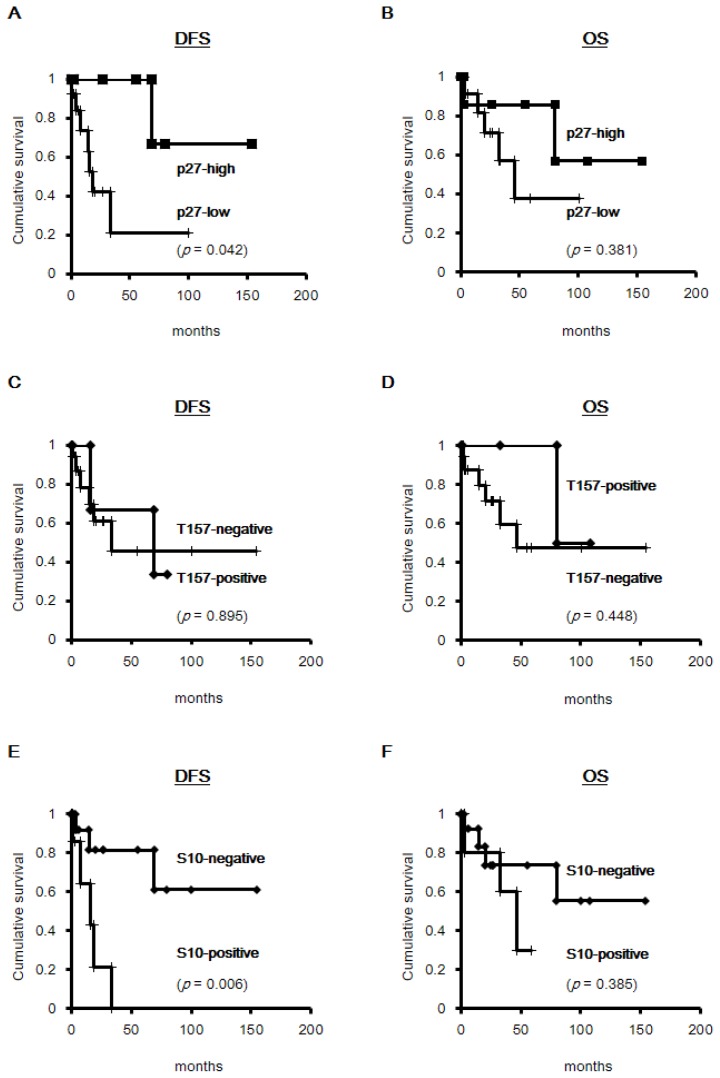
Kaplan-Meier curves for disease-free survival (DFS) and overall survival (OS) in HCC patients with NASH. (**A** and **B**) DFS and OS curves for patients according to p27 expression (p27-high, high-p27 expressers; p27-low, low-p27 expressers); (**C** and **D**) DFS and OS curves for patients according to phospho-p27 (T157) expression; (**E** and **F**) DFS and OS curves for patients according to phospho-p27 (S10) expression. *p*-values were obtained by the log-rank test.

To evaluate the clinical significance of p27, the prognosis of patients with NASH-related HCC was compared with their p27 status. Kaplan-Meier survival analysis showed that disease-free survival was 66.7% and 21.0% for the low- and high-p27 expressers, respectively, and decreased p27 expression was statistically associated with cancer recurrence (log-rank; *p =* 0.042; Figure 4A). Although statistical significance was not reached (*p =* 0.381), high-p27 expressers had a relatively better overall survival rate than low-p27 expressers (Figure 4B). Phosphorylation of p27 at T157 was not associated with disease-free survival (Figure 4C; *p =* 0.895) or overall survival (Figure 4D; *p =* 0.448); however, phosphorylation of p27 at S10 was observed to be significantly correlated with the prognosis of the patients. Kaplan-Meier survival analysis showed that disease-free survival was 61.1% and 0% in the groups with negative and positive expression of phosho-p27 (S10) (*p =* 0.006) (Figure 4E), respectively, and overall survival rates were 55.3% and 30% for negative and positive phospho-p27 (S10) expression, respectively (*p =* 0.385) (Figure 4F).

To further evaluate if either p27 or phospho-p27 (S10) could be an independent prognostic factor in NASH-related HCC, we investigated their association with recurrence-free rates within three years. A multivariate Cox regression model indicated that the levels of both p27 protein expression and phosphorylation at S10 failed to be independent predictors of cancer recurrence or death of the patients. Intriguingly, however, univariate analysis revealed that phospho-p27 (S10) was the only factor associated with increased cancer recurrence rates (hazard ratio 7.623 [1.457–39.882], *p =* 0.016) (Table 4). This was in contrast to the clinicopathological factors including tumor size (*p =* 0.092), intrahepatic metastasis (*p =* 0.980), histological grade (I *vs*. II) (*p =* 0.682), vascular invasion (*p =* 0.497), and tumor stage (pT1 *vs*. pT2-3) (*p =* 0.639), all of which were not correlated with cancer recurrence in the patients. Of note, cases with less than three years of DFS (*n =* 7) (Table 5) showed distinctive p27 and phospho-p27 expression profiles; p27 was decreased in six of seven cases (86%), while phospho-p27 (S10) was positive in five of seven cases (71%). In contrast, increased phospho-p27 (S10) was detected in only two cases (14%) in the case group without tumor recurrence (*n =* 14), suggesting that phospho-p27 (S10) might be correlated with tumor recurrence in NASH patients.

### 2.2. Discussion

NASH is a non-viral progressive liver disease characterized by hepatic steatosis and hepatocellular injury, which is often accompanied with insulin resistance and mitochondrial dysfunction [29,30]. Rising prevalence of NASH as well as NAFLD has drawn the attention of oncologists to individuals with obesity and diabetes mellitus, because accumulating evidence suggests that pathological features of NASH may contribute to HCC development [31]. Currently, however, the NASH scientific research field has a short history, and the exact epidemiology of cancer development in NASH is not still defined. Hui *et al*. [32] reported that the risk of HCC in NASH-associated liver cirrhosis was significantly lower than HCV-associated cirrhosis, and Sanyal *et al*. [33] reported that NASH had a significantly lower risk of HCC development as compared with HCV-associated cirrhosis (10/149 *vs*. 25/147 patients at risk; *p* < 0.01). In contrast, Ascha *et al*. [34] recently reported that the yearly cumulative incidence of HCC was 2.6% and 4.0% in patients with NASH cirrhosis and with HCV cirrhosis (*p =* 0.09), respectively, suggesting that the risk of cancer development is comparable between NASH- and HCV-associated cirrhosis. These lines of conflicting evidence may be partly because of the fact that the diagnosis of NASH can only be achieved by histological analysis, and the difficulty of diagnosing NASH at an early stage may mislead the clinicians from the real prevalence of this disease.

Recently, many studies have reported that patients with NASH-related HCC show a high rate of cancer recurrence. Ohki *et al*. [35] reported that the cumulative cancer recurrence rate at three years was 75.1% in NASH patients with high visceral fat, and Tokushige *et al*. [36] reported that cumulative cancer recurrence at five years was 69.8% in NASH-related HCC, which was comparable to HCV-associated HCC. Likewise, Reddy *et al*. [37] reported that there were no differences in recurrence-free survival between NASH and HCV groups (median, 60 *vs*. 56 months; *p =* 0.303). We recently examined the natural outcome of 17 NASH patients with surgically resected HCC, and found that cumulative disease-free survival at five years was 66% [5]. Although our reported data seem to be relatively favorable compared with previous reports, we found that cumulative disease-free survival of NASH-related HCC reached a plateau within two years after the resection [5]. Therefore, to improve the prognosis of NASH patients, investigating the early recurrence of HCC would be valuable.

To date, many studies using cultured cell systems and animal models have suggested that the cancer development in NASH may be governed by multiple hits of oncogenic events including insulin resistance, oxidative stress, lipid peroxidation, inflammatory cytokines, and autophagy [30,38]. In human studies, however, reports of cases of NASH-related HCC have been rare, and the number of tumors analyzed were limited to less than 22 cases [39–45]. Aiming to get quality data that would be sufficient for statistical analyses, we obtained clinical data and surgically resected tissue samples from a total of 22 cases of NASH-related HCC. Although the number of cases studied here seems rather small compared with previous studies of virus-associated HCC, we found that the obtained data were sufficient for statistical analyses.

The cell-cycle regulator p27 is a widely recognized adverse prognostic marker in various cancer types [10,14,18,19]. To address whether functional status of p27 is associated with tumor aggressiveness in NASH-related HCC, we examined the level of p27 expression and p27 phosphorylation at T157 and S10 by immunohistochemical analysis. Our analysis showed that p27 was decreased in 13 of 22 tumors (59%), and was significantly associated with enlarged tumor size and increased cell proliferation (*p =* 0.01 and *p <* 0.01, respectively). Although there have been no reports of p27 involvement in NASH-related HCC, previous studies have already reported that p27 is closely involved in tumor progression and prognosis in hepatitis virus-related HCC. Ito *et al*. first reported that p27 is an independent determinant of HCC recurrence among several G1-S cell-cycle regulators including pRb, p21, p16, p53, cyclin D1, and cyclin E [12], and accumulating evidence has now revealed that decreased p27 expression is a poor prognosis indicator of HCC [13,14,22]. Together with our data obtained in this study, these factors may support the idea that p27 is a critical prognostic indicator of HCC associated with hepatitis viruses or NASH. Although the initial step of carcinogenesis in NASH might be different from virus-associated cirrhosis to some extent, deregulated cell-cycle regulation might be a common mechanism of tumor aggressiveness in HCC. Given the findings that the PCNA labeling index was significantly increased in the low-p27 expresser subgroup in our study (*p =* 0.009), further investigation of cell-cycle regulators using a large number of tissue samples from NASH-related HCC is desirable.

In our study, we found that phospho-p27 (S10), but not phospho-p27 (T157), has a strong prognostic value in NASH-related HCC. Expression of phospho-p27 (S10) was correlated with an increased PCNA labeling index (*p* < 0.001), but was not correlated with any of the clinical parameters of the tumors such as tumor size (*p =* 0.510), vascular invasion (*p =* 0.476), histological grade (*p =* 0.349), or tumor stage (0.490). Nevertheless, Kaplan-Meier analysis with log-rank testing clearly showed that the disease-free rate was significantly decreased in the subgroup positive for phospho-p27 (S10). Univariate analysis indicated that phospho-p27 (S10) is only an independent risk for cancer recurrence within three years (*p =* 0.016). Very recently, phosphorylation of p27 at S10 has been shown to have a new role in cell migration. With mitogenic stimuli, p27 is phosphorylated at S10 and exported from the nucleus to bind actin in the cytoplasm. In this setting, p27 induces cell migration, which is far from its known ability to inhibit progression of the cell cycle [26,46]. Therefore, it is reasonable that phospho-p27 (S10) might be a unique, independent prognostic determinant of cancer cells. Although there have been no clinical studies of phospho-p27 (S10) in HCC, a close relationship between nuclear expression of phospho-p27 (S10) and advanced clinical stage has been recently reported in gliomas and ovarian cancers [47,48]. We are now investigating the prognostic significance of phospho-p27 (S10) in virus-associated HCC.

In this study, to address whether the level of p27 expression or phospho-p27 (S10) might be independent prognostic factors in NASH-related HCC, we attempted to perform a multivariate logistic regression analysis, but failed due to the limited number of the cases studied. We suggest performing further examination using a large number of patients and meta-analysis. NASH is increasingly becoming an indicator for liver transplantation due to HCC, and early diagnosis may help lower the instance of patients requiring transplants. Our study is the first to show a cell-cycle regulator as a possible biomarker for predicting the prognosis of NASH-related HCC. As the exact mechanism by which HCC develops in patients with NASH remains unclear, further studies using more clinical samples would be valuable to improve the prognosis of NASH patients.

## 3. Experimental Section

### 3.1. Study Subjects

For histological examination, HCC tissues along with matched non-tumorous tissue portions obtained from surgically resected subjects were fixed in formalin and embedded in paraffin. NASH diagnosis was performed according to the following criteria: (1) an absence of significant alcohol intake (ethanol consumption of less than 20 g/day); (2) histological features representing hepatic steatosis and inflammation with hepatocyte injury (ballooning), with or without fibrosis; and (3) exclusion of other liver diseases including viral hepatitis, autoimmune hepatitis, drug-induced hepatitis, primary sclerosing cholangitis, primary biliary cirrhosis, and metabolic disease such as Wilson’s disease and hemochromatosis [38,49]. The pathological diagnoses and analyses of HCC were made according to the General Rules for the Clinical and Pathological Study of Primary Liver Cancer [50]. Normal liver tissue samples were surgically obtained from three individuals without liver disease, and used for normal controls in immunohistochemical analysis. Written informed consent was obtained from all the human subjects included in the study under an institutional review board approved protocol, and the study protocol conformed to the ethical guidelines of the 1975 Declaration of Helsinki as reflected in *a priori* approval by the institution’s human research committee.

### 3.2. Immunohistochemistry

Deparaffined tissue sections were rehydrated in graded ethanol, and endogenous peroxidase activity was blocked using 0.3% peroxide in methanol. The sections were heated in a microwave in 10 mM sodium citrate (pH 6.5) for antigen denaturation, then incubated with a monoclonal mouse anti-human p27 antibody (1:100; SX53G8; DAKO, Cytomation, Glostrup, Denmark), a rabbit anti-phospho-p27 (T157) antibody (1:100; R & D Systems, Inc., Minneapolis, MN, USA), a rabbit anti-phospho-p27 (S10) antibody (1:100, Santa Cruz Biotechnology, Santa Cruz, CA, USA), or an anti-PCNA antibody (1:2000; PC10; DAKO, Cytomation, Glostrup, Denmark) at 4 °C overnight using the Vectastain Elite ABC Kit (Vector Laboratories, Burlingame, CA, USA). Control immunoglobulin was used as a negative control. Color development was performed using 3,3-diaminobenzidine (Sigma Chemical Co., St. Louis, MO, USA). Counterstaining was achieved by staining with hematoxylin.

L.I. of p27, phospho-p27 (T157), phospho-p27 (S10), and PCNA were determined as the percentage of immune-positive cells in 200 cells at five randomly selected fields. Immunostaining was evaluated by three independent observers, including one veterinary pathologist (T.W.), blinded to the clinical data for the histochemistry slides. According to previous studies [13,14,48], weak cytoplasmic staining of p27 was regarded as a non-specific reaction, and patients with staining below or above the cutoff value of 50% of the p27 L.I. were categorized as “low-p27” or “high-p27” expressers, respectively. Patients with less than 5% L.I. of phospho-p27 (T157) or (S10) were categorized into T157- or S10-negative groups, respectively, and those with greater than 5% L.I. of phospho-p27 (T157) or (S10) were categorized into T157- or S10-positive groups, respectively.

### 3.3. Western Blotting

Aliquots of tissue lysates (20 μg of protein) were obtained from four HCC samples with matched adjacent liver tissue samples from NASH patients, and from two normal liver tissue samples. Protein samples were separated by SDS-PAGE and transferred onto Immobilon-P membranes (Millipore, Bedford, MA, USA). Membranes were reacted with each of the antibodies used for immunohistochemical analysis, and probed with horseradish peroxidase-conjugated secondary antibody. Plots of protein were visualized using an enhanced ECL Western blotting detection system (GE Healthcare, Oslo, Norway). The band intensities were quantified using Image-J analysis software (version 1.44; NIH, Bethesda, MD, USA) and normalized by β-actin (Sigma Chemical Co., St. Louis, MO, USA) band intensity.

### 3.4. Statistical Analysis

The clinicopathological features of the HCC patients with different p27 and phospho-p27 (T157) expression levels were compared using the Spearman’s rank correlation coefficient analysis or the Fisher’s exact test. Correlation with cell proliferation and p27 status was analyzed using the Mann-Whitney test. Survival times were calculated from the data for curative surgery to HCC recurrence, death, or last follow-up of the patients using the Kaplan-Meier method, and the significance was evaluated by the log-rank test. The prognostic significance of clinical and pathological characteristics was determined using univariate and multivariate Cox regression analyses. All analyses were performed using SPSS software (Statistical Product and Service Solutions version 20; SPSS Inc., Chicago, IL, USA), and the differences were considered significant for values of *p* < 0.05.

## 4. Conclusions

Our obtained results show that a cell-cycle regulator, p27, known to be a valuable prognostic factor of hepatitis virus-associated HCC, can also be a useful biomarker to predict cancer recurrence of NASH-related HCC. Immunohistochemical analysis showed that decreased p27 expression was correlated with cell proliferation and size enlargement of the tumor, and a close link with a shortened cancer-free period was observed in the patients. Univariate analysis showed that phosphorylated p27 at S10 was the only indicator for a reduced cancer-free period in this disease. To date, there have been no clinical reports of cell-cycle regulators in NASH-related HCC. We propose that investigating the cell-cycle regulators might be of value for understanding the mechanism of cancer development and progression in the patients with NASH, which has been attracting worldwide attention along with the increased prevalence of obesity. To date, few studies using verified tissue samples from NASH-related HCC patients have been reported, and the limited number of samples available for analysis presents a major obstacle to investigations into the pathogenic process of this disease. Early diagnosis of HCC through close follow-up of NASH patients is desirable to improve our understanding of the molecular mechanisms of NASH-related HCC.

## Figures and Tables

**Table 1.** Clinicopathological profiles of non-alcoholic steatohepatitis (NASH)-related hepatocellular carcinoma (HCC).

Clinical characteristics	Patients	(%)
**Tumor size**		

≤5	15	68.2
>5	7	31.8

**Vascular invasion**		

absent	17	77.3
present	5	22.7

**Histological grade**		

I	6	27.3
II	16	72.7

**Tumor stage**		

pT1	14	63.6
pT2	5	22.7
pT3	3	13.6

**Tumor recurrence**		

absent	14	63.6
present	8	36.4

**Table 2.** Correlation of p27 and phosphorylated p27 expression in NASH-related HCC.

p27 phosphorylation		p27 expression			

		low (*n =* 13)	high (*n =* 9)	*r*	*p*
p-p27 (T157)	−	13	5		
	+	0	4	0.652	0.003

p-p27 (S10)	−	8	7		
	+	5	2	0.193	0.378

Data are determined by the Spearman’s coefficient test.

**Table 3.** Association between clinicopathological parameters and p27 in NASH-related HCC.

Tumor status	p27			phospho-p27 (T157)			phospho-p27 (S10)		

	low	high	*p*	−	+	*p*	−	+	*p*
Tumor size									

≤5	6	9		11	4		10	4	
>5	7	0	0.010	7	0	0.187	5	3	0.510

Vascular invasion									

absent	11	6		15	2		11	6	
present	2	3	0.316	3	2	0.209	4	1	0.476

Histological grade									

I	2	4		3	3		**5**	1	
II	11	5	0.155	15	1	0.045	10	6	0.349

Tumor stage									

pT1	10	4		13	1		9	5	
pT2-3	3	5	0.135	5	3	0.116	6	2	0.490

*p* values are estimated by the Fisher’s exact test.

**Table 4.** Univariate analysis of three-years disease-free survivals for NASH-related HCC.

Clinicopathological factors	HR	95% CI	*p*^a^
Tumor size (>5 cm)	4.109	0.795–21.217	0.092
Intrahepatic metastasis	1.021	0.197–5.297	0.980
Histological grade (II) a	1.415	0.269–7.445	0.682
Vascular invasion	1.768	0.341–9.157	0.497
Tumor stage (pT2-4) b	0.673	0.129–3.514	0.639
p27 (low-expressor)	6.211	0.734–52.581	0.094
phospho-p27 (T157)	0.530	0.063–4.449	0.559
phospho-p27 (S10)	7.623	1.457–39.882	0.016 *

a The hazards ratio is given as I *vs.* II in the Edmondson-Steiner grade;

b The hazards ratio is given as stage pT1 *vs*. pT2-4 defined by the General Rules for the Clinical and Pathological Study of Primary Liver Cancer adopting the tumor-node-metastasis classification;

* *p*< 0.05.

**Table 5.** Expression profiles of p27 and phosphor-p27 in the cases with less than three years of disease-free survival.

Cases a	Age	Gender	DFS b	p27	p-p27 (T157)	p-p27 (S10)
1	73	female	1.7	low	−	+
2	60	male	7.8	low	−	+
3	75	female	18.5	low	−	+
4	64	male	3.7	low	−	−
5	55	male	33.6	low	−	+
6	83	male	15.9	high	+	+
7	66	male	14.9	low	−	−

a Disease-free survival period was less than three years in seven cases;

b Disease-free survival period (months).
